# A new method for individual condylar osteotomy and repositioning guides used in patients with severe deformity secondary to condylar osteochondroma

**DOI:** 10.1186/s13023-021-01713-8

**Published:** 2021-01-30

**Authors:** Lei Qi, Ningning Cao, Weiwen Ge, Tengfei Jiang, Linfeng Fan, Lei Zhang

**Affiliations:** 1 Department of Oral and Cranio-Maxillofacial Surgery, Ninth People’s Hospital, Shanghai Jiao Tong University School of Medicine, Shanghai, 200011 China; 2 National Clinical Research Center for Oral Diseases, Shanghai, 200011 China; 3 Shanghai Key Laboratory of Stomatology and Shanghai Research Institute of Stomatology, Shanghai Jiao Tong University School of Medicine, Shanghai, 200011 China; 4 Department of Radiology, Ninth People’s Hospital, Shanghai Jiao Tong University School of Medicine, Shanghai, 200011 China; 5 Department of Oral and Maxillofacial Surgery, Ninth People’s Hospital, Shanghai Jiao Tong University School of Medicine, No. 639 Zhizaoju Road, Shanghai, 200011 China

**Keywords:** Condylar osteochondroma, Condylar repositioning guide, Condylar osteotomy guide, Deformity

## Abstract

**Background:**

Mandibular condylar osteochondroma (OC) could lead to facial morphologic and functional disturbances, such as facial asymmetry, malocclusion, and temporomandibular joint dysfunction. However, after condylar OC resection, the inaccurate reposition of the neocondyle still needs to be solved. The purpose of this study was to explore the feasibility of the condylar osteotomy and repositioning guide to reposition the neocondyle in the treatment of patients with severe deformity secondary to condylar OC.

**Results:**

Three patients with severe deformity secondary to OC of the mandibular condyle were enrolled in this study. With the aid of condylar osteotomy and repositioning guide, condylar OC resection and repositioning were carried out, and the accuracy and stability of these guides were evaluated. All patients healed uneventfully, and no facial nerve injury and condylar ankylosis occurred. Compared with the computerized tomography scans in centric relation before surgery and 3 days after surgery, the results showed that the facial symmetry was greatly improved in all the patients. Also, after the superimposition of the condylar segments before surgery and 3 days after surgery, the postoperative reconstructed condyles had a high degree of similarity to the reconstruction of the virtual surgical planning. Observed from the sagittal and coronal directions, the measurements of condylar positions were very close to those of virtual surgical planning. Moreover, it also showed stable results after a 1-year follow-up.

**Conclusions:**

For patients with severe deformity secondary to condylar OC, condylar osteotomy, and repositioning guide was expected to provide a new option for the improvement of facial symmetry and occlusal relationship.

## Introduction

Osteochondroma (OC), a kind of osteocartilaginous exostosis, is considered as the common tumor of long bones, comprising approximately 35 to 50% of all benign bone tumors [1, 2]. The condyle is the most vulnerable site in the facial bones [3]. In the process of the development period, as the growth center of the mandible, the condyle has an essential role in maintaining appearance and function [4]. Therefore, condylar OC could result in aesthetic deformities and functional disturbances, including facial asymmetry, unaligned dentition, occlusal malformation, and temporomandibular joint (TMJ) disorders [5].

In order to improve the aesthetics and function, different treatment methods are selected according to the degree of dental and maxillofacial deformities secondary to condylar OC. At present, condylar OC resection combined with orthognathic surgery, including mandibular sagittal split ramus osteotomies (SSRO), Le Fort I maxillary osteotomies, and genioplasties is the most frequently used treatment methods [6]. For condylar OC resection, the preauricular incision alone or in combination with cervical incisions is the most common option [7]. Yet, this approach is associated with an increased risk of facial nerve injury, and it also tends to leave the scar on the face [8]. Recently, the awareness of minimally invasive surgical techniques to replace traditional larger procedures has attracted interest from an increasing number of surgeons and patients. With endoscopy, direct visual access, along with the capability for illumination and magnification allows for minimal tissue dissection and improved surgical fields [9]. Compared to other fields, the use of endoscopic techniques for exposure, resection, and reconstruction of the condylar disorders are relatively novel approaches. Our previous study demonstrated that endoscope-assisted condylar OC resection combined with simultaneous contralateral SSRO could be chosen in the treatment of asymmetric prognathism secondary to condylar OC [10]. The retrospective study also showed that the combination of conservative condylectomy via the intraoral approach based on the endoscope and simultaneous contralateral SSRO is effective in improving the facial symmetry in the treatment of the condylar OC [11].

For patients with more severe maxillary and mandibular deviation and worse malocclusion, intraoral condylectomy and contralateral SSRO could not be used to place the residual neocondyle into the fossa to restore the occlusal relationship and TMJ function. To correct the deformities and improve the function, bilateral SSRO must be performed intraorally. Unfortunately, during the follow-up process, we found that this technique may cause ischemic absorption or necrosis of the proximal bone segments of the condylectomy side due to excessive periosteum decollement.

Considering the problems mentioned above, for patients with severe asymmetric deformities secondary to condylar OC, we chose a preauricular incision to remove the condylar OC and then performed orthognathic surgery to avoid this situation. Different from the conventional preauricular incision, we innovatively applied the condyle osteotomy and repositioning guides to ensure the accurate position of residual neocondyle with respect to the fossa. Also, the preliminary evaluation was made to investigate its accuracy and stability. This approach could provide a new option for the treatment of severe asymmetric deformities secondary to condylar OC.

## Materials and methods

### Patients

**Fig. 1:**
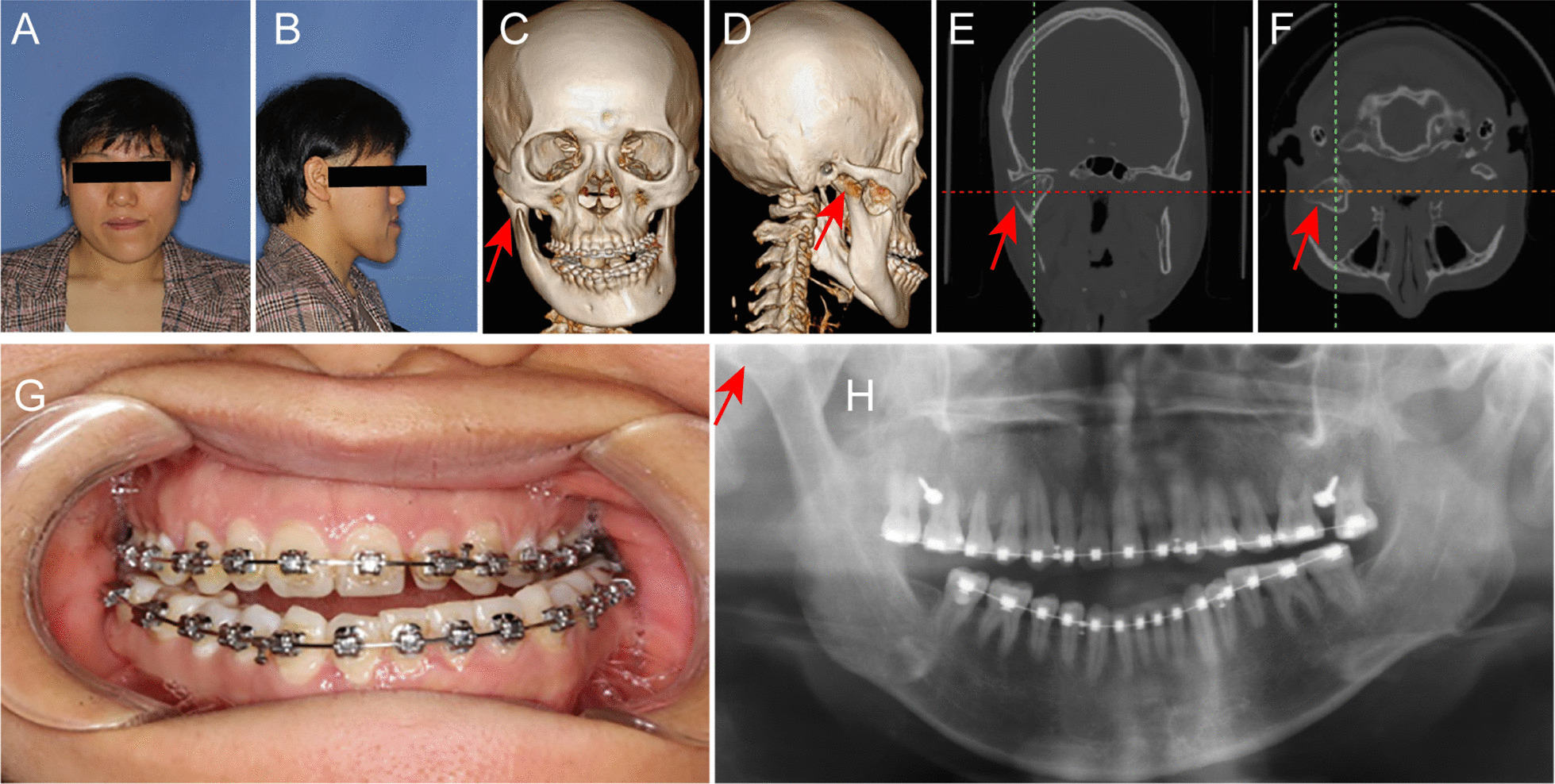
A woman with mandibular condylar osteochondroma. **a** Front profile; **b** lateral profile; **c** front three-dimensional CT image; **d** lateral three-dimensional CT image; **e** coronal CT scan; **f** horizontal CT scan; **g** intraoral view; **h** panoramic radiograph. Red arrow, condylar osteochondroma

Three patients with condylar OC accompanying dental maxillofacial deformities were enrolled in this preliminary study at the Department of Oral and Craniomaxillofacial Surgery of Shanghai Ninth People’s Hospital, Shanghai Jiao Tong University School of Medicine. Following clinical and radiographic examinations, the patients had no other craniomaxillofacial skeletal syndrome, traumas, or previous orthognathic surgery (Fig. 1). All the patients performed TMJ MRI before surgery to assess the position and condition of the articular disc. Then, all the patients underwent mandibular condylectomy of the affected side, and bilateral sagittal split ramus osteotomy (BSSRO). All operations were performed by the same surgical team with extensive experience in the field of orthognathic surgery. This study was approved by the Ethics Committee of Shanghai Ninth People’s Hospital, Shanghai Jiao Tong University School of Medicine. All patients were informed of the technology preoperatively and gave written consent to participation.

### Preoperative virtual surgical planning

**Fig. 2:**
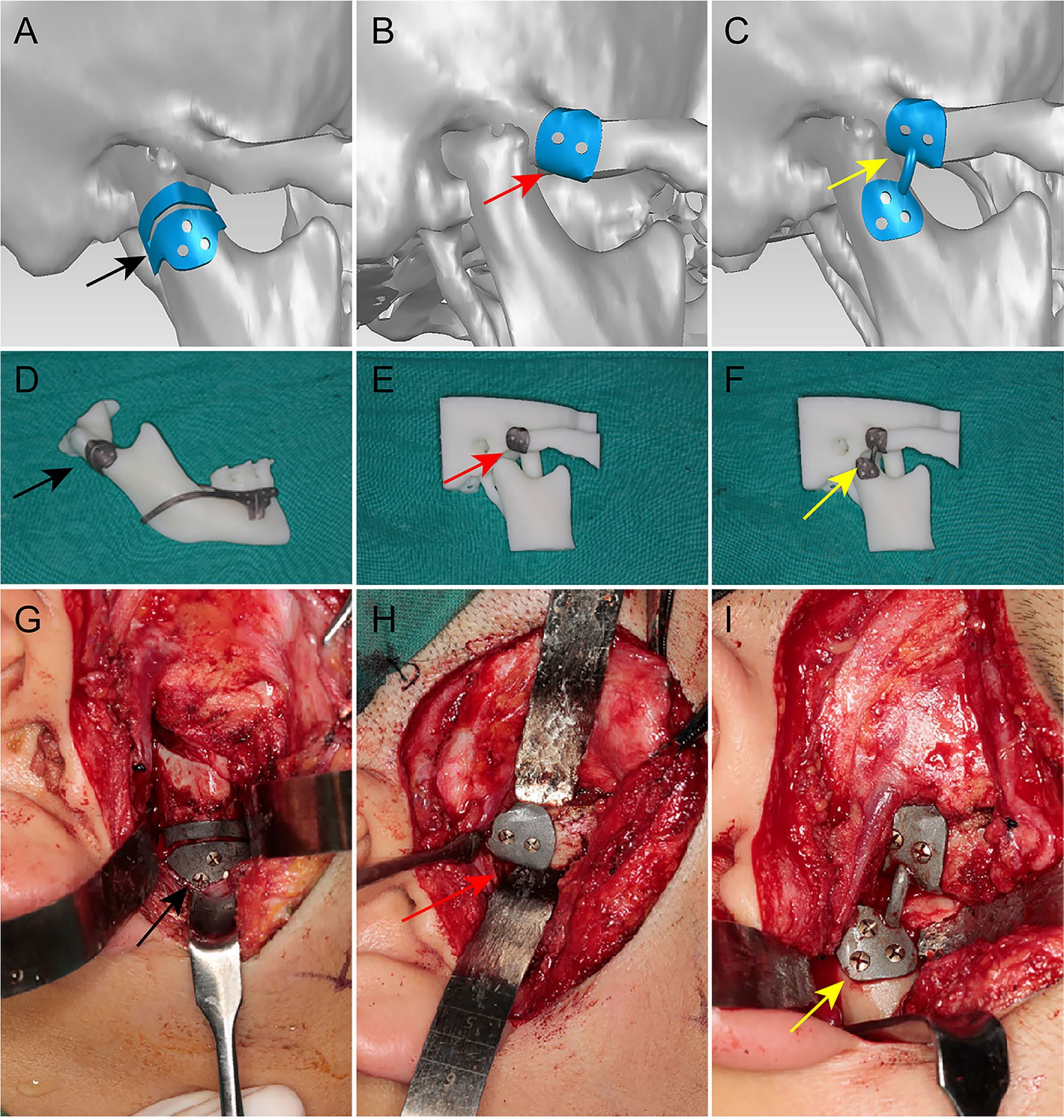
The guides in virtual design, stereomodel, and surgery. **a** Virtual condylar osteotomy guide; **b** virtual nail hole positioning guide at the root of the zygomatic arch; **c** condylar repositioning guide; **d** three-dimensional printed condylar osteotomy guide; **e** three-dimensional printed nail hole positioning guide; **f** three-dimensional printed condylar repositioning guide; **g** condylar osteotomy guide during surgery; **h** nail hole positioning guide during surgery; **i** condylar repositioning guide during surgery. Black arrow, condylar osteotomy guide; red arrow, nail hole positioning guide; yellow arrow, condylar repositioning guide

Preoperatively, the high-resolution computed tomography (CT) of the craniomaxillofacial skeleton was taken by a GE Medical System LightSpeed CT scanner (GE Healthcare, Buckinghamshire, UK). All two-dimensional CT images were saved in digital imaging and communications in medicine (DICOM) format and imported into SurgiCase CMF 5.0 (Materialise, NV Leuven, Belgium) for the reconstruction of three-dimensional virtual models of the craniomaxillofacial skeleton. After considering the chief complaint, virtual surgical planning and simulation were created by the surgical team and 3D design engineer according to the clinical physical examinations and three-dimensional malformation analyses. The osteotomies and condylar OC resection were performed virtually by the surgeons so as to achieve an optimal aesthetic appearance. Preoperative virtual condyle osteotomy and repositioning guides were used to ensure accurate positioning of the neocondyle (Fig. 2a–c). The patients’ neocondyle was placed into the mandibular joint fossa to recreate shape and position using individual condylar repositioning guides. The mirror image of the unaffected condyle was obtained by using the central plane as the reference position so as to ensure the symmetry during the process of virtual reconstruction.

### Design and construction of condylar guides

The data of craniomaxillofacial skeleton bone were imported into Unigraphics NX 7.5 (Siemens PLM Software, TX, USA) for designing of the condylar and other templates. Using the pre-simulated data, skull, maxillary and mandibular stereomodels, condylar osteotomy guide, condylar repositioning guides, and maxillary osteotomy and repositioning guides were manufactured. Condylar guides included condylar osteotomy guide and condylar repositioning guide. The condylar osteotomy guide embraced the neck of the condyle. The position of the condylar osteotomy guide was designed according to the size and position of condylar OC to ensure complete tumor resection. Under the guidance of the osteotomy guide, the osteotomy line was exactly the same as the virtual design. After condylar OC resection, the remaining three holes were placed on the residual bone for the use of a zygomatic-borne condylar repositioning guide. The condylar repositioning guide was divided into two parts: nail hole positioning guide at the root of the zygomatic arch and the zygomatic-borne condylar repositioning guide. The nail hole positioning guide was embraced at the root of the zygomatic arch according to pre-determined virtual design, and the position was unique to ensure the accuracy of the following condylar repositioning. Two holes were also placed on the zygomatic arch for the use of a zygomatic-borne condylar repositioning guide. The zygomatic-borne condylar repositioning guide was fixed on the stationary zygomatic bone. Also, the neocondyle was repositioned using the remaining three holes on the neck of the condyle and two holes on the root of the zygomatic arch. The five holes for fixation screws were carefully placed to avoid damaging the essential tissues and organs while fixing the screws. The zygomatic-borne condylar repositioning guide helped the surgeons to determine the unique optimal site quickly and accurately.

The mandible is unstable due to the presence of condylar OC. Hence, the movement of the maxilla was controlled using maxillary osteotomy and repositioning guides to ensure the stability and accuracy of operation. All the guides were made using the three-dimensional printing technique before the operation, and all were tried on the three-dimensional skull, maxillary and mandibular stereomodels to ensure that the guides were placed smoothly (Fig. 2d–f). After this, the guides were sterilized for use during operation.

### Surgical technique

The surgery was performed by one team. After adequate exposure of the condylar OC and the neck of the condyle, the articular disc was peeled and perserved intactly without damaging the peripheral anatomic structures. Then, the condylar osteotomy guide was secured to the neck of condyle through designed holes. During the operation, special care was taken to protect the important surrounding tissues, including facial nerve. The osteotomies were performed by referring to the cutting slots to make the condylar OC resection as effectively as in virtual planning (Fig. 2g). After removing the osteotomy guide, the three remaining nail holes were reserved for the condyle repositioning guides. Then, after the root of the zygomatic arch was also exposed, the nail hole positioning guide was screwed on the bone through predrilled holes (Fig. 2h). Since the uniqueness of the guide plate contour, the guide plate tightly embraced the root of the zygomatic arch, and the position of the nail hole was also unique. After removing the guide, the two remaining holes were also reserved for the condyle repositioning guides.

The zygomatic-borne condylar repositioning guide was placed on the bone of the zygomatic arch and the neck of condyle along the holes left by the osteotomy guide and the nail hole positioning guide (Fig. 2i). The fit between the inside surface of the guide and the outside surface of the bone was excellent. The spatial movement of the condyle was structurally determined by the repositioning guides according to the virtual design. The repositioning guide was used to achieve the optimal position of neocondyle through the predrilled holes. After determining the position of the neocondyle, the articular disc was sutured to the articular capsule to envelope the neocondyle.

Due to the instability of condyle, the maxillary movement was determined using 3D printed osteotomy and repositioning guides, after which the surgeon performed BSSRO and repositioned the mandibular segment in the traditional manner to correct the facial asymmetry.

### Postoperative validation and follow-up

A craniomaxillofacial CT scan was taken for each patient at 3 days (T1) and 1 year (T2) postoperatively. The accuracy of the condyle position was assessed by comparing the actual postoperative operation to the virtual planned one (T0). Both postoperative and planned CT data were imported into SurgiCase CMF 5.0, and the deviations were measured by Geomagic Studio 2013 software. Deviations were defined using the following indicators: (1) 3D deviation of the osteotomy, (2) 3D deviation of the condylar segments at 3 days postoperatively. In order to quantitatively compare the accuracy, the following measurements were recorded from the two-dimensional CT images of T0 and T1. For the coronal image, the medial joint space (MJS) and lateral joint space (LJS) were measured from the most prominent medial and lateral condylar points to the glenoid fossa. For the sagittal image, the anterior joint space (AJS), superior joint space (SJS), and posterior joint space (PJS) were measured from the most prominent anterior, posterior, and superior condylar points to the glenoid fossa. The linear measurements of the affected joint spaces in the actual postoperative and virtual planned models were assessed and compared with each other.

To observe the stability and accuracy of the condyle repositioning guides, the postoperative CT data (T2) and 3D deviation of the condylar segments were obtained at 1 year postoperatively. The two-dimensional CT images were also acquired to compare the relevant linear indicators mentioned above. The functional and esthetic outcomes were also assessed based on the postoperative dental occlusion, images and clinical examination.

## Results

**Fig. 3:**
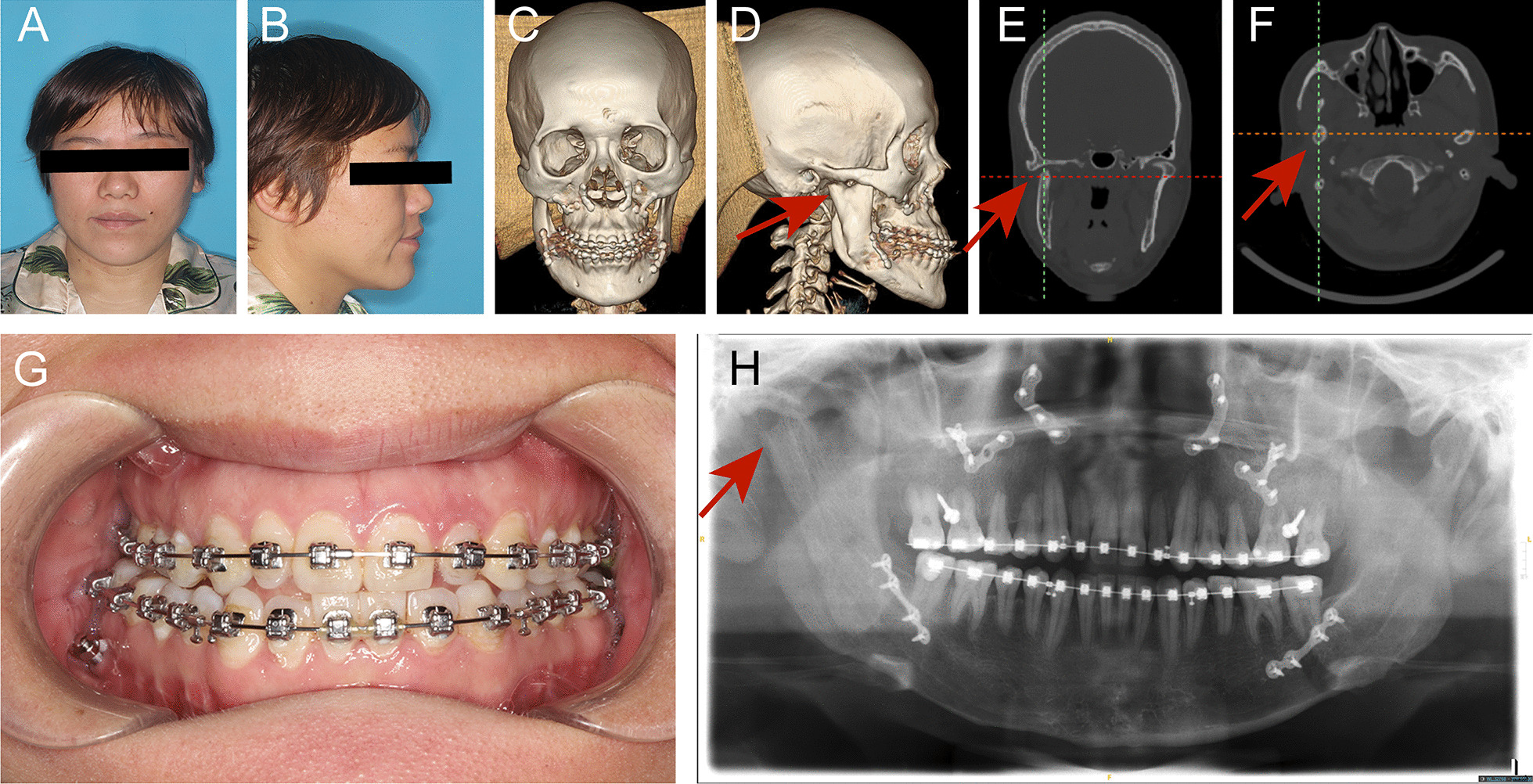
Data collection at 1 year after surgery. **a** front profile; **b** lateral profile; **c** front three-dimensional CT image; **d** lateral three-dimensional CT image; **e** coronal CT scan; **f** horizontal CT scan; **g** intraoral view; **h** panoramic radiograph. Red arrow, neocondyle

The new computer-assisted surgery was performed on three consecutive patients (1 male and 2 Females) who required condylar OC resection and orthognathic surgery. The articular discs of the patients in our study were intact with no displacement, rupture, or perforation through observing TMJ MRI before surgery (Additional file 1: figure 1). All operations were successful. There were no signs of unpredictable use of the guides, facial nerve trauma, articular clicking and no pain, or other wound-related complications. Based on the findings from the clinical examination and 3D CT, there was no evidence of tumor recurrence in any of the patients, and all the patients achieved good occlusion and symmetric contour (Fig. 3).

### Evaluation of the accuracy of condylar osteotomy and repositioning guide

**Fig. 4:**
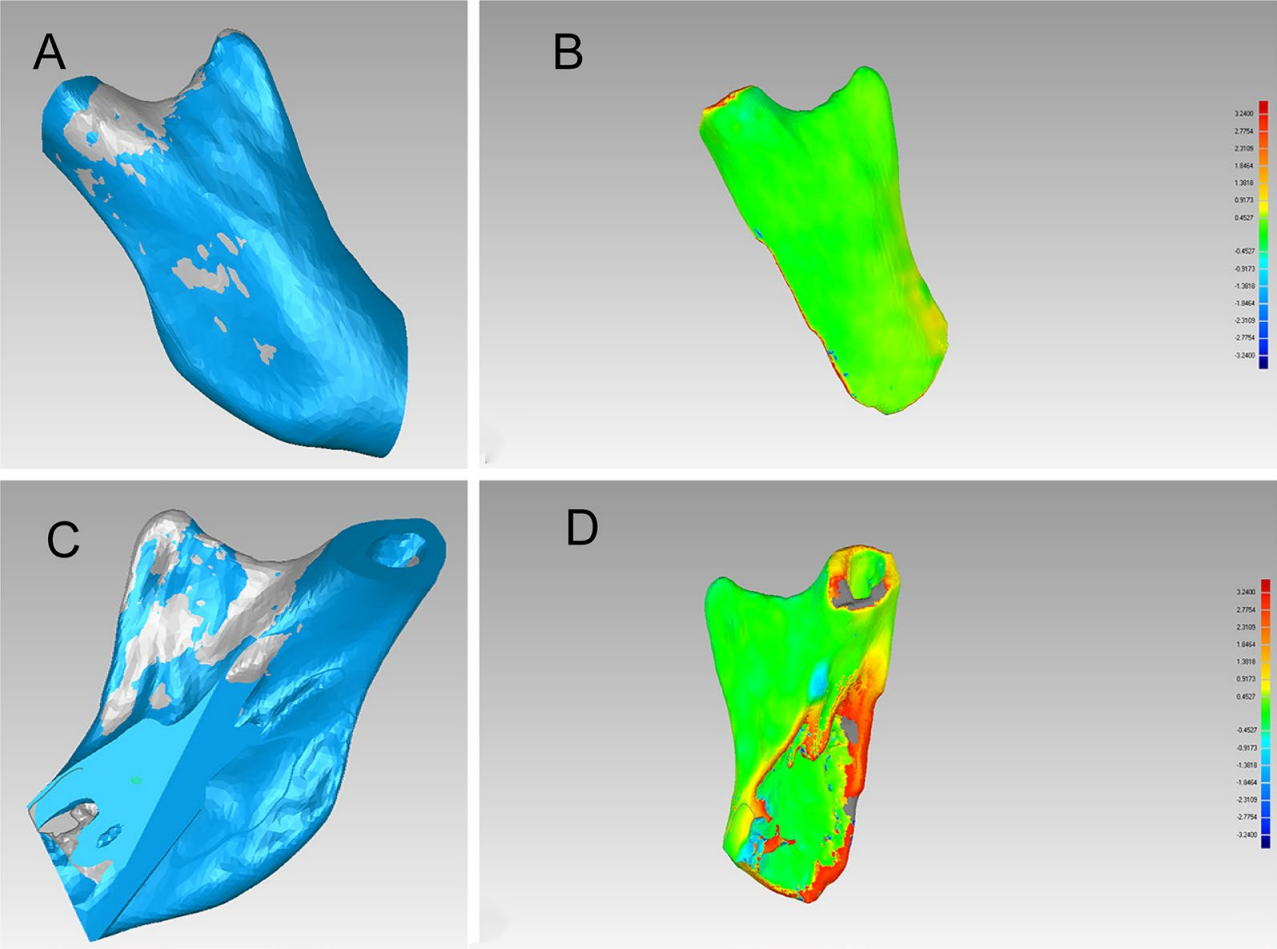
Evaluation of the accuracy of condylar osteotomy guide. **a**, **c** Superimposition of the virtual plan (blue) and postoperative CT scan at 3 days after surgery (grey); **b**, **d** the postoperative accuracy study of condylar osteotomy guide using the Geomagic Studio 2013 software

**Fig. 5:**
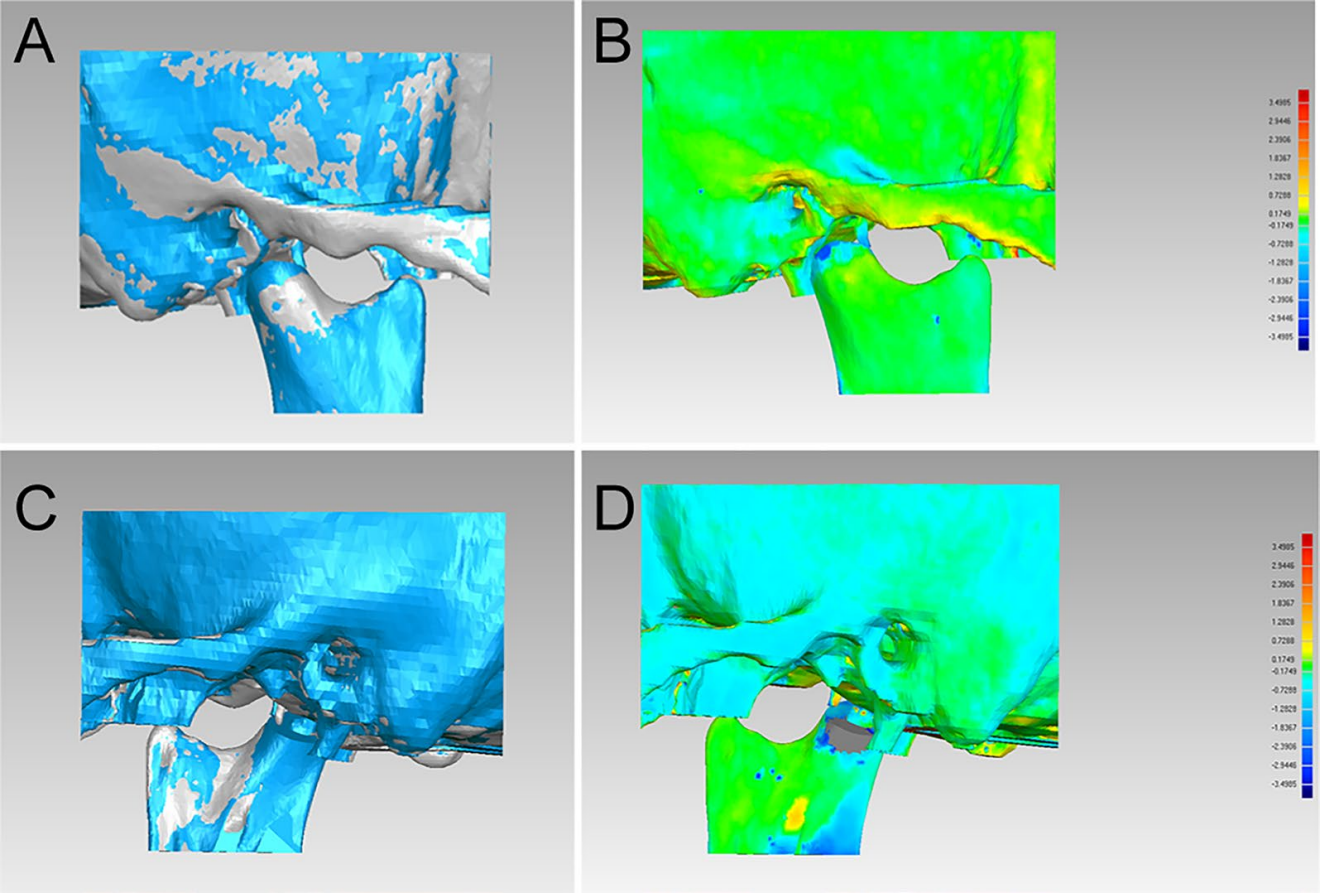
Evaluation of the accuracy of condylar repositioning guide. **a**, **c** superimposition of the virtual plan (blue) and postoperative CT scan at 3 days after surgery (grey); **b**, **d** the postoperative accuracy study of condylar repositioning guide using the Geomagic Studio 2013 software. **e**, **f** the magnification view of part of the zygomatic arch, fossa, and neocondyle

After the superimposition of the condylar segments of T0 and T1, the postoperative reconstructed condyles had a high degree of similarity to the reconstruction of the virtual surgical planning (Fig. 4). The 3D deviations between T0 and T1 are shown in Table 1. To condylar ostotomy guide, the mean 3D deviation of the condylar segments compared with the virtual ones was 0.41 mm; the maximum 3D deviation, 3.24 mm; the standard deviation, 1.00 mm. The position of the postoperative reconstructed condyle and the reconstructive ones of the virtual surgical planning were compared to evaluate the accuracy of the condylar repositioning guides (Fig. 5). To condylar repositioning guide, the mean 3D deviation of the skull and condylar segments at 3 days after surgery compared with the virtual ones was 0.42 mm; the maximum 3D deviation, 3.50 mm; the standard deviation, 0.58 mm (Table 1). The mean data of the condylar position and the standard deviation are shown in Table 2. Observed from the sagittal and coronal directions, the data of T1 was very close to the T0 (Table 2).

**Table 1:** Postoperative accuracy study of condylar repositioning with osteotomy and repositioning guide

	Osteotomy guide	Repositioning guide	
	T1 vs T0	T1 vs T0	T2 vs T1
Mean 3-D (mm)	0.4101	0.4176	0.8143
Max 3-D (mm)	3.2400	3.4985	4.4987
Standard deviation(mm)	1.0036	0.5770	1.0146
RMS estimate (mm)	1.1010	0.6045	1.0298

T0, preoperative design; T1, 3 days after surgery; T2, 1 year after surgery; D:deviation; RMS, root mean square

**Table 2:** Measurement of the condylar position using condylar repositioning guide in the coronal, sagittal at T0, T1 and T2

	T0 (mean ± SD)	T1 (mean ± SD)	T2 (mean ± SD)
AJS	3.34 ± 0.33	3.37 ± 0.32	4.19 ± 0.44
SJS	6.81 ± 0.19	6.81 ± 0.20	8.11 ± 0.41
PJS	4.46 ± 0.12	4.41 ± 0.10	6.15 ± 0.10
MJS	5.79 ± 0.56	5.80 ± 0.56	6.07 ± 0.46
LJS	3.31 ± 0.10	3.35 ± 0.19	4.28 ± 0.77

AJS, anterior joint space; SJS, superior joint space; PJS, posterior joint space; MJS, medial joint space; LJS, lateral joint space; T0, preoperative design; T1, 3 days after surgery; T2, 1 year after surger; SD, standard deviation

### Evaluation of the stability of condylar repositioning guide

**Fig. 6:**
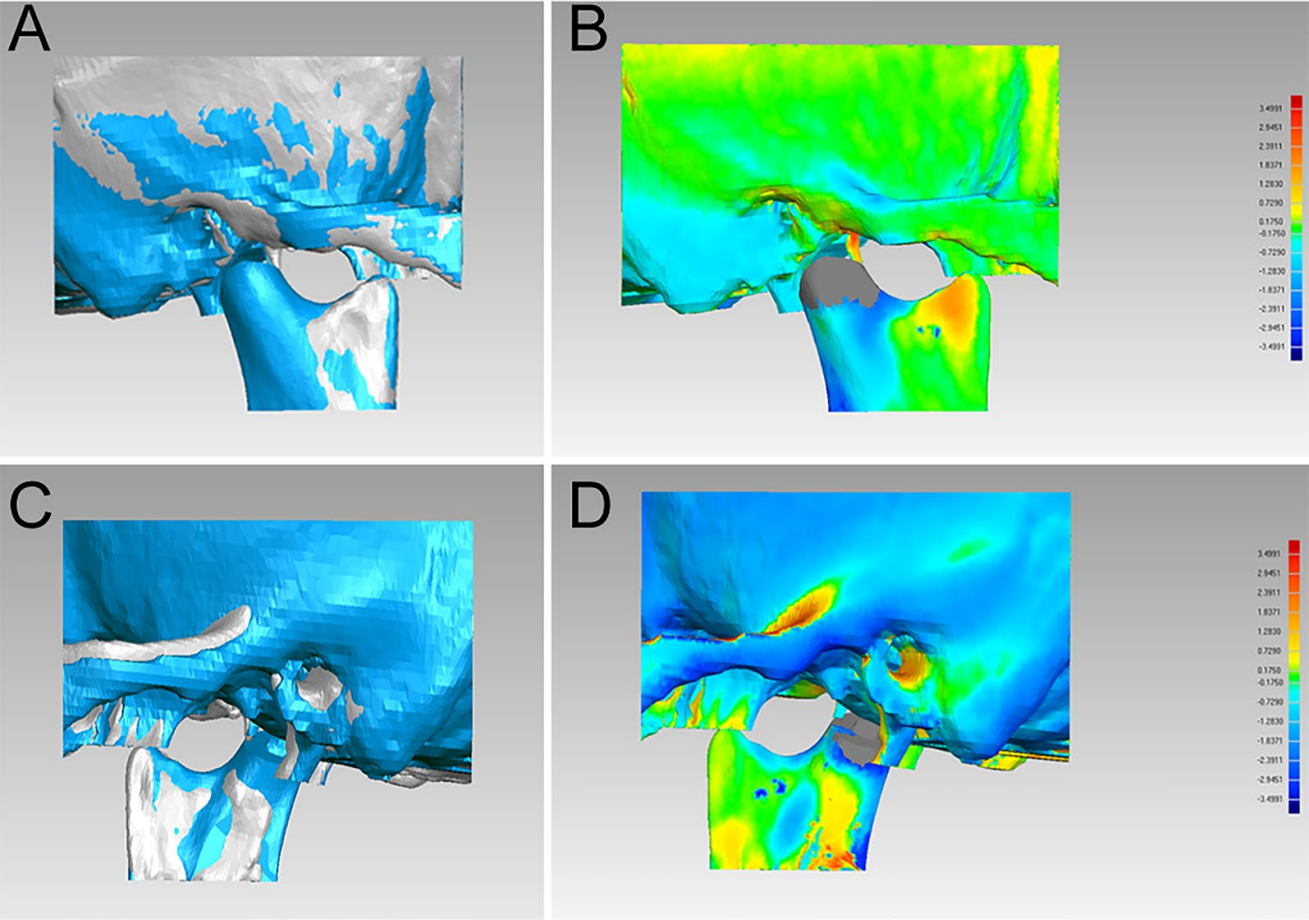
Evaluation of the stability of condylar repositioning guide. **a**, **c** superimposition of postoperative CT scan at 3 days after surgery (grey) and postoperative CT scan at 1 year after surgery (blue); **b**, **d** the postoperative stability study of condylar repositioning guide using the Geomagic Studio 2013 software. **e**, **f** the magnification view of part of zygomatic arch, fossa, and neocondyle

To evaluate the stability of condylar position with condylar repositioning guides, the skull and condylar segments were compared with the preoperative virtual ones 1 year after surgery (Fig. 6). The 3D deviation between T1 and T2 were also shown in Table 1. The mean 3D deviation of the postoperative skull and condylar segments compared with the virtual ones was 0.81 mm; the maximum 3D deviation, 4.50 mm; the standard deviation, 1.01 mm (Table 1). The mean data of the condylar position and the standard deviation of T2 are also shown in Table 2. Compared with T1, the condylar measurement indexes of T2 in all dimensions had some alterations (Table 2).

## Discussion

The complications of condylar OC vary with the size of the tumor and its main growth direction [12, 13]. The main clinical features include loss of condylar function, progressively increased facial asymmetry, prognathic deviation of the chin, open bite on the affected side, and cross-bite on the contralateral side [14]. Therefore, the goals of condylar OC treatment include surgical resection of the tumor, correction of facial asymmetry, and malocclusion [15]. After condylar OC resection, the position of neocondyle is crucial for maintaining postoperative symmetry facial contours and the stability of the occlusal relationship. Different surgical techniques for the treatment of condylar OC have been used based on different conditions including condylectomy, orthognathic surgery, and TMJ reconstruction surgery [16, 17]. Condylectomy can be categorized as high condylectomy, low condylectomy, proportional condylectomy [18]. High condylectomy was performed when the resection is performed within 5 mm from the upper side of the condylar head. In low condylectomy, removal of the growth centre and simultaneous preservation of condylar neck could correct the vertical excess. A proportional condylectomy was performed when the resection volume was adjusted for each individual. It is worth noting that the condylectomy must completely remove the tumor, while ensuring that the remaining mandibular ramus is as high as the contralateral side to the great extent, and leaving a certain gap between the neocondyle and the joint fossa to prevent the occurrence of ankylosis.

Condylectomy or accompanying joint sacrifice and subsequent condylar reconstruction via extraoral incision have been reported in many studies [19–21]. Yet, the conventional preauricular incision has certain disadvantages, including facial nerve injury, articular capsule exposure, and permanent scarring [22]. Moreover, the treatment of complete condylectomy and subsequent autogenous or alloplastic TMJ reconstruction remains controversial [19, 23]. Over recent years, the aesthetics requirements have gradually increased while restoring the function. The application of minimally invasive incisions during surgery has attracted increased attention from many surgeons and patients.

To achieve the goals mentioned above, individual surgical planning is indispensable. Computed tomography and computer-assisted design could be used to diagnose the lesion, to perform surgical simulation, and to evaluate the accuracy of the technology. With the aid of an endoscope, the condylar OC could be exposed and resected via an intraoral approach [24, 25]. Removing only a portion of the condylar head and reshaping of the residual condylar stump as neocondyle could be successfully performed with the help of intraoperative endoscopic navigation, preoperative virtual surgical planning, and simulation [26]. A previous study showed that this joint preservation approach combined with orthognathic surgery, including contralateral SSRO and Le Fort I maxillary osteotomy, led to an accurate and stable outcome [27]. The endoscope-assisted condylar OC resection intraorally offers great advantages with no significant complications such as facial nerve injury and scarring compared with conventional extraoral incisions [28]. Nevertheless, for patients with severe deformity, endoscope-assisted condylar OC resection combined with contralateral SSRO could not place the residual neocondyle into the fossa. Hence, in addition to endoscope-assisted condylar OC resection, the BSSRO is required to restore the positional relationship between neocondyle and fossa. However, these complicated and numerous operations are performed intraorally, which may cause excessive periosteum decollement. This condition may, in turn, result in the ischemic absorption and even necrosis of the proximal bone segment.

In view of these complications, for patients with severe deformity, the preauricular incision, condylar OC resection, and BSSRO via intraoral incision are necessary. Besides these approaches, maxillary Le Fort I osteotomy or genioplasty or lower border osteotomy might be required in different situations to achieve the goal of correcting deformities and malocclusion [29]. These surgical approaches could avoid the occurrence of ischemic absorption and necrosis of the proximal bone segment. However, one of the major difficulties related to this surgical method is the repositioning of the residual condylar stump [30]. Repositioning of neocondyle on the proximal segment after condylar OC resection and orthognathic surgery is a critical factor influencing the maintenance of facial symmetry, stable occlusal relationship, and TMJ function [31]. Postoperative changes in the neocondyle position with respect to the glenoid fossa could lead to multiple undesirable effects, including internal derangement of the TMJ, loss of mandible contour, neocondyle absorption, and loss or reduction in occlusion and mastication [32].

In the present study, we presented a novel condylar osteotomy guide and condylar repositioning guide to solve these problems. In order to overcome the instability of the mandible, maxillary 3D-printed osteotomy and repositioning guides were also simultaneously used to perform orthognathic surgery. Our study demonstrated that this technique could be used to ensure the correct positioning of the neocondyle into fossa after condylar OC resection. The postoperative follow-up results have also shown that the technique could provide clinically acceptable accuracy and stability in repositioning the neocondyle. These guides were designed and made using the CAD/CAM technique based on three-dimensional planning. The condylar osteotomy guide in our surgery could completely guide the condylar OC resection and reduce the additional surgical time and damage caused by repeated grinding. The condylar repositioning guide placed on the predrilled holes of the osteotomy guide could be used for the optimal positioning of the neocondyle with respect to the fossa. The newly designed guides were placed on the fixed zygomatic arch, which eliminated the potential errors caused by the movement of the maxilla or mandible during surgery. Preoperative virtual planning decreased intraoperative changes to ensure the optimal position relationship between neocondyle and fossa and avoid damage that could be caused by anatomical occurrences that were not considered preoperatively. Superimposition of the virtual segments and the actual segments after computer-assisted surgery showed that the mean 3D deviation of the condylar osteotomy guide between T0 and T1 was 0.41 mm; furthermore, the mean 3D deviation of condylar repositioning guide between T0 and T1 was 0.42 mm. The follow-up results demonstrated that the mean 3D deviation of the condylar repositioning guide between T1 and T2 was 0.81 mm. Thus, there is a high degree of similarity between the reconstructed condylar segments and the virtual planned ones. To further evaluate the accuracy and stability of this novel condylar repositioning guide, the distances between the most prominent points to the glenoid fossa in the coronal and sagittal CT images were measured. The results showed that the data of T1 were very close to that of T0 observed from the sagittal and coronal directions. However, compared with the phase of T0 and T1, the condylar position in all dimensions of T2 showed some alterations. However, the facial symmetry and occlusion relationship did not significantly change in any of the patients. It is known that the position and shape of condyle tend to gradually change after surgery [33]. For the patients, the position and shape of the neocondyle obtained after condylar OC resection may change with the reconstruction of the occlusal relationship and the adaptation of the neonatal condyle [34]. The continual adaptation of position and shape may be beneficial to the maintenance of condylar function, facial symmetry, and occlusal relationship [35].

The approach presented in the current study showed certain advantages. First, for patients with severe deformities secondary to condylar OC, the most significant advantage was the use of condylar repositioning guide, which could accurately restore the optimal relationship between neocondyle and fossa. The position and stability of neocondyle were important for maintaining the TMJ function, facial symmetry, and occlusion relationship. Moreover, the design of extraoral incision could avoid ischemic absorption and even necrosis of the proximal bone segment after removing the condylar OC. Another advantage of this computer-assisted surgery was that the condylar osteotomy guide and the repositioning guide used for condyle osteotomies and reposition neocondyle respectively provided a duplication of the preoperative virtual surgical plan and eliminated the need for intraoperative measurement, thus substantially saving time. However, the present study also had some limitations, including the small sample size and short follow-up time, which should be addressed by future studies. Also, the biggest concern related to this technique was extraoral incision resulting in facial nerve injury and scarring. In this study, none of the patients showed any signs of facial nerve injury in the follow-up.

Currently, the use of condylar osteotomy and repositioning guide combined with orthognathic surgery in the treatment of asymmetric prognathism caused by condylar OC is still in its infancy. Nevertheless, this surgical technique offers great advantages with no significant complications. The secondary deformities and malocclusion can be corrected by simultaneous orthognathic surgery. Therefore, condylar osteotomy guide and repositioning guide used for condyle OC resection and reposition neocondyle extraorally combined with orthognathic surgery intraorally provide a valuable option for patients with severe jaw deformities secondary to condylar OC.

## Supplementary Information

**Additional file 1: Figure 1.** The evaluation of position and condition through TMJ MRI before surgery. A, B: The TMJ MRI for sagittal view of condylar OC and normal side. C, D: The TMJ MRI for coronal view of condylar OC and normal side.

## Data Availability

The supporting datasets analyzed during the current study are available from the corresponding author on reasonable request.

## Abbreviations

OC: Osteochondroma
TMJ: Temporomandibular joint
SSRO: Sagittal split ramus osteotomies
BSSRO: Bilateral sagittal split ramus osteotomy
CT: Computed tomography
DICOM: Digital imaging and communications in medicine
