# Supplementary figures and images for: A new method for individual condylar osteotomy and repositioning guides used in patients with severe deformity secondary to condylar osteochondroma
Source: Orphanet J Rare Dis. 2021 Jan 30;16:59. doi: 10.1186/s13023-021-01713-8 (PMC7847561; doi:10.1186/s13023-021-01713-8)

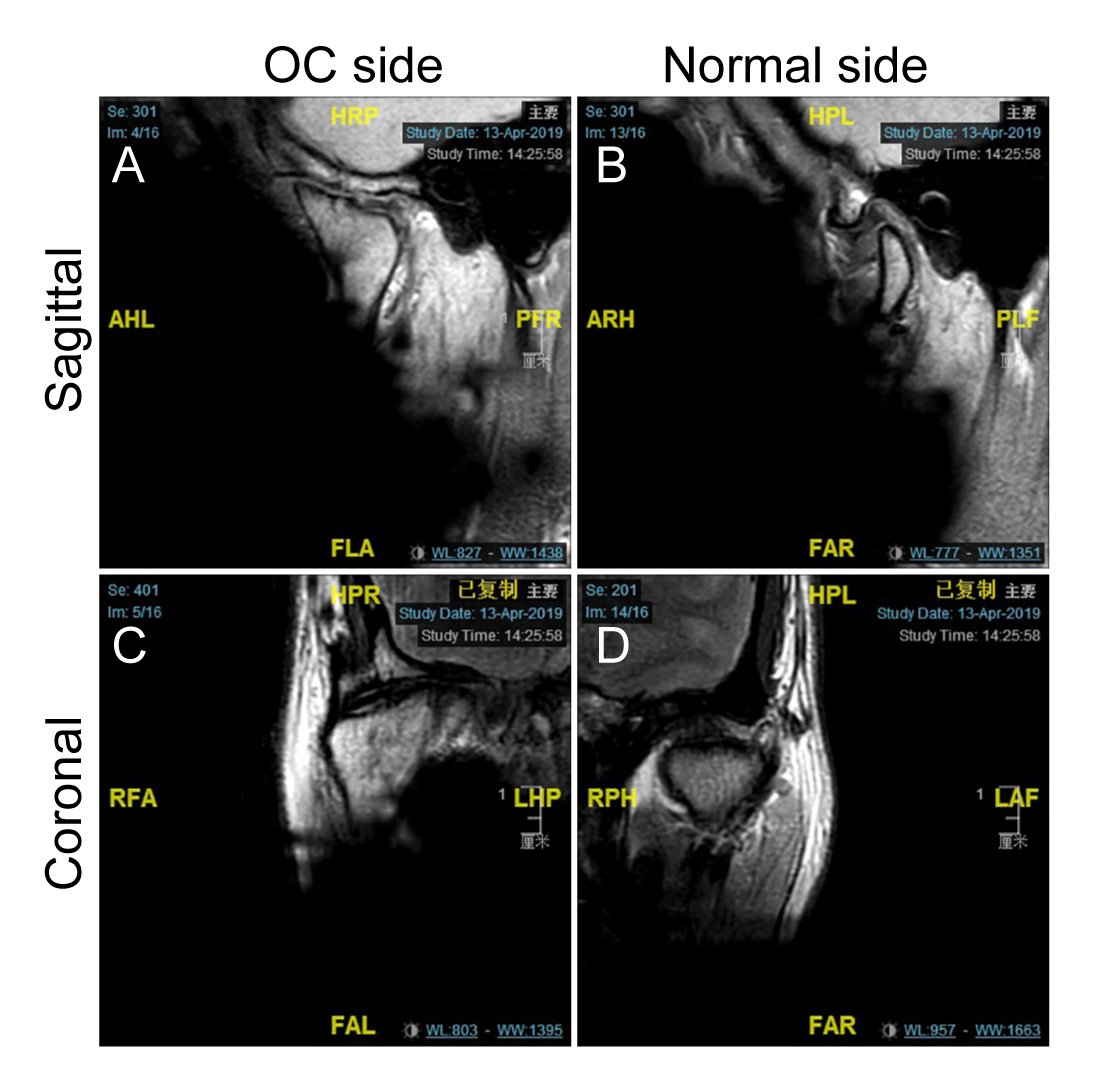

Supplement: Supplementary file 1 — Additional file 1: Figure 1. The evaluation of position and condition through TMJ MRI before surgery. A, B: The TMJ MRI for sagittal view of condylar OC and normal side. C, D: The TMJ MRI for coronal view of condylar OC and normal side. [file 13023_2021_1713_MOESM1_ESM.tif]
